# Acid α-glucosidase (GAA) activity and glycogen content in muscle biopsy specimens of patients with Pompe disease: A systematic review

**DOI:** 10.1016/j.ymgmr.2024.101085

**Published:** 2024-04-25

**Authors:** Benedikt Schoser, Nina Raben, Fatbardha Varfaj, Mark Walzer, Antonio Toscano

**Affiliations:** a Friedrich-Baur-Institute, Department of Neurology, LMU Klinikum, Ludwig-Maximilians University, Munich, Germany; b M6P Therapeutics, St. Louis, MO, USA; c Astellas Pharma Global Development, Inc., Northbrook, IL, USA; d ERN-NMD Center of Messina for Neuromuscular Disorders, Department of Clinical and Experimental Medicine, University of Messina, Messina, Italy

**Keywords:** Pompe disease, Skeletal muscle biopsy, Acid α-glucosidase, Glycogen

## Abstract

Pompe disease is a rare genetic disorder characterized by a deficiency of acid α-glucosidase (GAA), leading to the accumulation of glycogen in various tissues, especially in skeletal muscles. The disease manifests as a large spectrum of phenotypes from infantile-onset Pompe disease (IOPD) to late-onset Pompe disease (LOPD), depending on the age of symptoms onset. Quantifying GAA activity and glycogen content in skeletal muscle provides important information about the disease severity. However, the distribution of GAA and glycogen levels in skeletal muscles from healthy individuals and those impacted by Pompe disease remains poorly understood, and there is currently no universally accepted standard assay for GAA activity measurement. This systematic literature review aims to provide an overview of the available information on GAA activity and glycogen content levels in skeletal muscle biopsies from patients with Pompe disease.

A structured review of PubMed and Google Scholar literature (with the latter used to check that no additional publications were identified) was conducted to identify peer-reviewed publications on glycogen storage disease type II [MeSH term] + GAA, protein human (supplementary concept), Pompe, muscle; and muscle, acid alpha-glucosidase. A limit of English language was applied. Results were grouped by methodologies used to quantify GAA activity and glycogen content in skeletal muscle. The search and selection strategy were devised and carried out in line with Preferred Reporting of Items in Systematic Reviews and Meta-Analysis guidelines and documented using a flowchart. Bibliographies of papers included in the analysis were reviewed and applicable publications not already identified in the search were included.

Of the 158 articles retrieved, 24 (comprising >100 muscle biopsies from >100 patients) were included in the analysis, with four different assays. Analysis revealed that patients with IOPD exhibited markedly lower GAA activity in skeletal muscles than those with LOPD, regardless of the measurement method employed. Additionally, patients with IOPD had notably higher glycogen content levels in skeletal muscles than those with LOPD. In general, however, it was difficult to fully characterize GAA activity because of the different methods used. The findings underscore the challenges in the interpretation and comparison of the results across studies because of the substantial methodological variations. There is a need to establish standardized reference ranges of GAA activity and glycogen content in healthy individuals and in Pompe disease patients based on globally standardized methods to improve comparability and reliability in assessing this rare disease.

## 1 Introduction

Pompe disease, or glycogen storage disease type II (GSDII; OMIM #232300), is a rare autosomal recessive disorder caused by biallelic mutations in the acid α-glucosidase (GAA) gene (MIM 606800) that encodes the lysosomal enzyme acid α-1,4-glucosidase (GAA; EC 3.2.1.20), formerly known as acid maltase [1]. GAA is active in lysosomes, where it breaks down glycogen [1] and releases free glucose [2], which is then transported to the cytosol for use in various cellular pathways. Without GAA, excess lysosomal glycogen blocks autophagy, leading to the accumulation of autophagic debris that is detrimental to myofiber function [[3], [4], [5], [6], [7], [8], [9], [10]]. It is thought that a failure of productive autophagy and accumulation of potentially toxic ubiquitinated proteins contribute to severe muscle weakness and atrophy [11,12], the hallmarks of Pompe disease.

The severity of Pompe disease is largely determined by the levels of residual GAA activity and is broadly categorized as a continuous spectrum from infantile to juvenile and adult-onset forms. Infantile-onset Pompe disease (IOPD) is caused by a near complete loss of GAA activity (<1%), with the effects typically observed in the first year or even in the first days after birth. IOPD is characterized by generalized muscle weakness, severe muscle hypotonia, hypertrophic cardiomyopathy, and respiratory insufficiency [13]. Without treatment, patients with IOPD usually die by 1 year of age, primarily due to cardiac or respiratory failure [13,14]. Symptoms of late-onset Pompe disease (LOPD) vary greatly according to residual GAA activity (up to approximately −30% of normal). LOPD (alternatively classified as childhood, juvenile, or adult Pompe disease) encompasses patients with onset of symptoms from 12 months of age through adulthood and can manifest as late as the seventh decade of life [1]. LOPD typically progresses to both significant motor disability and respiratory insufficiency [3,4], the latter being the most life-threatening manifestation and the leading cause of morbidity in Pompe disease [1,5]. Unlike IOPD, hypertrophic cardiomyopathy is not typically observed in LOPD.

The current standard of care for Pompe disease is enzyme replacement therapy (ERT), comprising repeated intravenous infusions of a recombinant human GAA (rhGAA) precursor enzyme (alglucosidase alfa, avalglucosidase alfa, or cipaglucosidase alfa with miglustat), which were first approved in 2006, 2022, and 2023, respectively. ERT acts by replacing GAA and, although it can be disease modifying, it is not curative [[6], [7], [8], [9]]. In IOPD, ERT has been reported to rescue cardiac function, reduce the need for invasive ventilation, and improve survival [10,13]; however, in the long term (>30 months), IOPD patients still show disease progression with different muscular patterns and severe disease manifestations in the central nervous system, including cognitive problems [12,15,16]. In patients with LOPD, the response to ERT varies widely, and its effects can diminish over time [17]. Overall, the therapy has reduced mortality and improved mobility and respiratory function [10,18]; however, many patients still require wheelchairs and assisted ventilation despite ERT [10,[19], [20], [21]].

Although there are approaches that may improve the efficacy of ERT (e.g., increasing dose/frequency, improving uptake, upregulating expression of CI-M6PR or a combination of ERT with pharmacological chaperones to improve enzyme stability in plasma [[22], [23], [24]]), other modes of treatment, such as gene therapy (already in development [24]) may prove to be closer to a cure (or may prove to be more efficient). Gene therapy aims to replace the affected gene via a one-time infusion that results in continuous production of GAA [10,[25], [26], [27]].

Regardless of the approaches taken to modify the progression of Pompe disease, quantifying GAA activity and glycogen content in skeletal muscle provides information on the severity of the disease, which in turn guides treatment choices. Additionally, assessing these variables may help evaluate the response to therapy. However, there needs to be a greater understanding of GAA distribution and glycogen content in skeletal muscles from healthy individuals and those impacted by Pompe disease. Furthermore, there is currently no standard assay for GAA activity measurement that is universally agreed upon. This systematic literature review provides a detailed overview of the available information on the amount of GAA activity and glycogen content levels in skeletal muscle biopsy specimens from patients with Pompe disease.

## 2 Methods

### 2.1 Study design

The protocol for this systematic review was designed a priori and is reported by the Preferred Reporting of Items in Systematic Reviews and Meta-Analysis (PRISMA) statement [28] and the Recommendations for the Conduct, Reporting, Editing and Publication of Scholarly Work in Medical Journals from the ICMJE [29]. The initial literature search was performed on July 25, 2022, using PubMed, and Google Scholar on July 14, 2023, as reported in the PRISMA flow diagram. The search terms were as follows: 1) glycogen storage disease type II [MeSH term] + GAA, protein human (supplementary concept); 2) Pompe, muscle; and 3) Muscle, acid alpha-glucosidase.

#### 2.1.1 Search and selection strategy

**Fig. 1:**
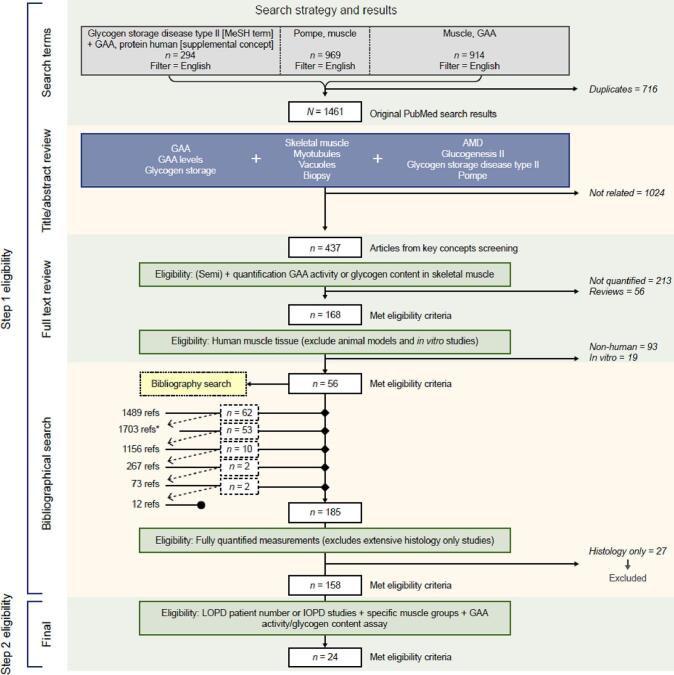
PRISMA flow diagram. The flow chart includes one review article that was used for the bibliography review, but then subsequenctly removed from the analysis. GAA: acid α-glucosidase; IOPD: infantile-onset Pompe disease; LOPD: late-onset Pompe disease.

The search and selection strategy were documented using a flowchart based on the PRISMA reporting standards [28] (Fig. 1). The identified literature was filtered using a stepwise approach.

More details on the selection process can be found in the **supplementary methods (Appendix A)**. Once the initial searches were complete, a bibliographic review of included articles was conducted, and relevant publications were included in a database to be analyzed. Two reviewers collaboratively collected data manually from each included report and created a database to hold the results. Strategies to ensure a comprehensive body of evidence included the use of broad search terms and subsequent review of reference lists from selected publications.

#### 2.1.2 Validation

The specificity of the search process was validated using an article (N. Raben et al. [30]) chosen before the analysis. This publication was selected because the topic was directly related to our study objective and, as it describes a murine model, it would initially be included and then excluded in the human muscle tissue criteria step, and fully quantified measurement step. In addition, the word “biopsy” was not in the title or abstract but was in the full-text study details. As such, if this article appeared in the final output, it was clear that the search criteria were not specific enough.

#### 2.1.3 Analysis

The studies were grouped into three “substrate groups” (the maltose group, the glycogen group, and the MuαG group) based on the assay substrates used to quantify GAA activity.

GAA activity levels from patients with the same disease subtypes and same muscle groups in each study were presented as median and range, unless they were reported as other specific formats in the original studies (i.e., mean ± SD).

## 3 Results

### 3.1 Search selection and study characteristics

The PRISMA flow chart outlines the overall search strategy and results (Fig. 1). All articles that remained after the searches and refinement were considered relevant for inclusion in the summary and analysis of GAA activity and glycogen content. A total of 168 articles were retrieved and subsequently reviewed in step 1 selection, which was narrowed down to 24 articles after the completion of step 2. A summary of all articles included in the analysis can be found in the supplementary table (Appendix B). The earliest article that met inclusion criteria was published in 1965, with the most recent study published online in 2018. An overview of the studies included is provided in Table 1.

**Table 1:** GAA activity and glycogen content levels in the reviewed studies (GAA activity unit: nmol of substrate hydrolyzed/min/g tissue).

Assay	Author, year	Patient number/ biopsy number	Muscle	pH	GAA activity			Glycogen content		
					nmol (substrate hydrolyzed)/min/g tissue			%		
					Median (range)	Test number	Control (mean ± SD or range)	Median (range)	Test number	Control (mean ± SD or range)
**Maltose**	Zellweger, 1965 [31]	1 (I)/2	Unknown	pH 4.5	5.5 (5.0–6.0)	2	28.0–110.0	1.2 (1.0–1.4)	2	NA
				pH 6.9	13.5 (11.0–16.0)	2	15.0–45.0			
	Zellweger, 1965 [31]	1 (L)/2	Ti	pH 4.5	5	1	28.0–110.0	0.8^§^	1	NA
				pH 6.9	14	1	15.0–45.0			
	Mekanik, 1966 [32]	1 (I)/2	G	NA	4.2	1	23.0–47.0	6.62^††^	1	0.12–1.53
	Engel, 1970 [33]	2 (I)/2	G	NA	–	–	–	11	1	0.85 (0.45–1.3)
	Engel, 1970 [33]	2 (I)/2	Q	NA	–	–	–	8.3	1	0.85 (0.45–1.3)
		4 (L)/5	Q	NA	32 (25–34)	3	425 ± 77	3.1 (2.7–3.5)	2	0.85 (0.45–1.3)
							(28–826)			
	Engel, 1970 [33]	4 (L)/5	Tr	NA	4.9	1	425 ± 77	5.2	1	0.85 (0.45–1.3)
							(28–826)			
	Engel, 1970 [33]	4 (L)/5	Sartorius	NA	–	–	–	1.9	1	0.85 (0.45–1.3)
	Engel, 1970 [33]	4 (L)/5	Adductor	NA	–	–	–	1.1	1	0.85 (0.45–1.3)
	Pongratz, 1976 [34]	1 (I)/2	G	NA	0	1	NA	4.66	1	≤2
	Pellegrini, 1978 [35]	1 (I)/3	B	NA	0	1	NA	12.4	1	0.4–1.1
	Read, 2001 [36]	5 (L)/9	Unknown	NA	7.15 (4.4–13.4)	4	114.4 ± 23.3	–	–	–
							(61.1–150.9)			
**Glycogen**	Slonim, 2006 [37]	2 (I)/3	Q	pH 4.0	6.45 (3.00–9.89)	2	27.3 ± 5.45	3.12 (0.83–5.41)	2	1.03 ± 0.18
				pH 6.5	19.85 (19.32–20.38)	2	19.32			
**MuαG**	van den Hout, 2001 & 2004^†^ [38,39]	4 (I)/4	Q	pH 4.0	3.9 (2.5–6.2)	4	133.3–666.7	209.6 (74.7–369.0)	8	3.0–18.0
	Klinge, 2005^†^ [40,41]	2 (I)/2	Q	pH 4.3	BDL	2	10 ± 5.55	6.65 (6.3–7.0)	2	≤1.5
	Slonim, 2006 [37]	2 (I)/3	Q	pH 4.0	3.33 (1.67–5)	2	45.17 ± 11.39	3.12 (0.83–5.41)	2	1.03 ± 0.18
				pH 6.5	63.61 (51.11–76.11)	2	72.22 ± 19.83			
	Kishnani, 2006 [11]	6 (I)/6	Q	pH 4.3	<0.055^‡^	6	NA	–	–	–
	Kishnani, 2007 [42]	18 (I)/18	Q	NA	0.02 (0.0–0.15)^¶^	18	NA	0.12–1.13	18	NA
	Koeberl, 2018 [43]	7 (L)/7 Treatment	Q	NA	140	6	NA	0.75	7	NA
		4 (L)/4 Control	Q	NA	200	4	NA	0.73	4	NA

B: biceps; BDL: below limit of detection; G: gastrocnemius; GAA: acid α-glucosidase; I: infantile-onset Pompe disease; L: late-onset Pompe disease; MUαG: 4-methylumbelliferyl α-glucopyranoside; NA: not available; Q: quadriceps; Ti: tibialis; Tr: triceps.

Some individuals provided more than one biopsy.

^†^These studies reported data from the same patients.

^‡^GAA activity was tested with both maltose and glycogen as substrates in this study.

^§^Glycogen content level was within normal range.

^††^Unit: gram glucose/100 g tissue.

^¶^The result value was reported as mean (range) in the study.

Retrieved publications covered over 100 biopsies from over 100 patients. Several articles reported on the same cohort; in all instances, this is denoted as footnotes in the respective table.

### 3.2 Methods used to quantify GAA activity and glycogen content

GAA activity levels (ranges in case of multiple biopsies) and glycogen content levels (ranges in case of multiple biopsies) in each study are summarized and categorized based on the units of GAA activity level in Table 1, Table 2, Table 3, Table 4.

Among the 24 studies included, nine reported GAA activity measured using maltose as substrate, four reported GAA activity measured using glycogen as substrate, 10 reported GAA activity measured using MuαG as substrate, and three studies reported GAA activity measured using maltose/MuαG as substrate. One study reported GAA activity using both maltose and glycogen as substrates, and one reported GAA activity using both glycogen and MuαG as substrates.

### 3.3 GAA activity and glycogen content in muscle biopsies from healthy individuals

GAA activity levels in healthy individuals varied by the method used; there were no clear patterns based on the analysis method, pH, or muscle biopsied. GAA activity in healthy individuals was reported in eight of the 14 studies in which GAA activity was measured using substrate hydrolyzed/min/g tissue. When maltose was used as a substrate (*n* = 5 studies; *n* = 8 data sets), values ranged from 15.0 to 826.0 nmol maltose hydrolyzed/min/g tissue in biopsies taken from gastrocnemius (*n* = 1), quadriceps (*n* = 1), triceps (*n* = 1), tibialis (*n* = 2) and unknown (*n* = 2; Table 1); glycogen was used as a substrate in one study (two data sets from quadriceps biopsies), GAA activity values ranged from 19.32 to 27.3 nmol glycogen hydrolyzed/min/g tissue. GAA activity was measured using MuαG as a substrate in six studies (*n* = 8 data sets). However, only four studies (*n* = 4 data sets) reported data for healthy individuals; values ranged from 10 to 666.7 nmol MuαG hydrolyzed/min/g tissue – all biopsies originated from quadriceps (Table 1).

In the six studies where GAA activity was assessed using nmol/min/g protein, five studies provided GAA activity values in healthy individuals. One study used glycogen as the substrate and noted a control value of 113 nmol/min/g protein in a biopsy originating from an unspecified muscle group; two (*n* = 3 data sets) used MuαG as a substrate and noted results ranging from 20 to 399 nmol/min/g protein in quadricep biopsies, while three used maltose/MuαG as the substrate and recorded values ranging from 31.4 to 173.4 nmol/min/g protein in quadricep and unspecified biopsies (Table 2).

**Table 2:** GAA activity and glycogen content levels in the reviewed studies (GAA activity unit: nmol/min/g protein).

Assay	Author, year	Patient number/ biopsy number	Muscle	pH	GAA activity			Glycogen content		
					nmol/min/g protein			%		
					Median (range)	Test number	Control	Median (range)	Test number	Control
							(mean ± SD or range)			(mean ± SD or range)
**Glycogen**	Ripolone, 2018 [44]	17 (L)/17	Unknown	NA	8.45 ± 3.22^‡^		113 ± 41	–	–	–
**MUαG**	Verity, 1991 [45]	1 (I)/1	Q	pH 4.0	<0.3^§^	1	35–70	108	1	<10
				pH 6.5	130^§^	1	20–80			
	Amalfitano, 2001 [46]	3 (I)/3	Q	NA	6.83 (1.67–11.17)	3	399 ± 144	5.68	3	0.94 ± 0.55
								(5.13–5.90)		
**Maltose/ MUαG**	Angelini, 2003 & 2004^†^ [47,48]	5 (L)/5	Q	NA	1.8^¶^	1	90^¶^	–	–	–
	Nicolino, 2009 [49]	18 (I)/18	Q	NA	12.5 ± 11.0^║^	18	NA	7.1 ± 2.5	18	NA
	Angelini, 2003 & 2004^†^ [47,48]	5 (L)/5	Unknown	NA	2^¶^	1	31.4–173.4^¶^	–	–	–
		5 (L)/5	Unknown	NA	5 (0.9–8.42)^¶^	3	31.4–173.4^¶^	–	–	–

GAA: acid α-glucosidase; I: infantile-onset Pompe disease; L: late-onset Pompe disease; MUαG: 4-methylumbelliferyl α-glucopyranoside; NA: not available; Q: quadriceps.

^†^These studies reported data from the same patients.

^§^Unit: nmol/min/g non collagen protein.

^║^The result value was reported as mean (range) in the study.

^¶^Unit: picomol/min/g non collagen protein.

Three studies reported GAA activities using units of μM of glucose/min/g protein. Two (*n* = 4 data sets) used maltose as a substrate, with values ranging from 0.72 to 21.39 μM of glucose/min/g protein, and one study (*n* = 1 data set) used glycogen as the substrate and reported values of 0.58–1.83 μM of glucose/min/g protein from an unspecified muscle group (Table 3). Only one study used units of mol/g wet-weight muscle; mean GAA activity in healthy individuals was 8.03 ± 1.48 mol/g wet-weight muscle from an unspecified muscle group (Table 3).

**Table 3:** GAA activity and glycogen content levels in the reviewed studies (GAA activity unit: μM/min/g protein or mol/g of wet weight muscle).

Assay	Author, year	Patient number/ biopsy number	Muscle	pH	GAA activity			Glycogen content		
					μM of glucose/min/g protein			μg/mg protein		
					Value	Test number	Control (range)	Value	Test number	Control (range)
**Maltose**	Koster, 1978 & Busch, 1979† [50,51]	1 (I)/1	Unknown	pH 4.0	0.44	1	4.20–21.39	1480	1	32.1–79.5
				pH 6.5	2.89	1	0.72–4.11			
		1 (L)/2	Unknown	pH 4.0	0.56	1	4.20–21.39	86.4	1	32.1–79.5
				pH 6.5	1.03	1	0.72–4.11			
	Loonen, 1981^‡§^ [52]	1 (I)/1	Unknown	NA	0.44	1	4.29–21.39	1480	1	32–80
		1 (L)/2	Unknown	NA	0.69	1	4.29–21.39	86	1	32–80
**Glycogen**	Loonen, 1981‡§ [52]	1 (L)/2	Unknown	NA	0.09	1	0.58–1.83	86	1	32–80
					GAA Activity mol/g wet-weight muscle					
					Median (range)	Test number	Control (mean ± SD)			
**Maltose/ MUαG**	Angelini, 2003 & 2004† [47,48]	2 (L)/2	Unknown	NA	0.33 (0.33–0.33)	2	8.03 ± 1.48			

GAA: acid α-glucosidase; I: infantile-onset Pompe disease; L: late-onset Pompe disease; MUαG: 4-methylumbelliferyl α-glucopyranoside; NA: not available.

Some individuals provided more than one biopsy.

† These studies reported data from the same patients.

‡ Same patients as Koster, 1978 & Busch, 1979. However, the GAA activity result of the LOPD patient was different.

§ GAA activity was tested with both maltose and glycogen as substrates in this study.

Unlike the quantification of GAA activity, only two methods were used for glycogen quantification across all studies – % or μg/mg protein. Of the studies included in the analysis, nine reported glycogen values for healthy individuals ranging from 0.12% to 18% (Table 1, Table 2). Three studies reported values as μg/mg protein, with values ranging from 32 to 80 μg/mg protein (Table 3).

### 3.4 GAA activity and glycogen content in muscle biopsy specimens of patients with IOPD and LOPD

As with healthy individuals, biopsies from patients with either IOPD or LOPD originated from various muscles – biceps, gastrocnemius, quadriceps, tibialis, triceps, or an unspecified source.

As expected, GAA muscle activity was lower in patients with Pompe disease than in healthy individuals. In addition, GAA activity was lower in patients with IOPD compared with LOPD. In assays that reported values as nmol (substrate hydrolyzed)/min/g tissue using maltose as a substrate, ranged from 0 to 4.2 nmol maltose/min/g tissue for IOPD (*n* = 3 data sets; Table 1) and 4.4 to 34 nmol maltose/min/g tissue for LOPD (*n* = 5 data sets). Where glycogen was used as a substrate, values were reported as 3.0–20.38 nmol/glycogen/min/g tissue for IOPD (*n* = 2 data sets); no values were reported for LOPD. Assays using MuαG as a substrate reported 0–76.11 nmol/MuαG/min/g tissue for IOPD (*n* = 6 data sets) and 140–200 nmol/MuαG/min/g tissue for LOPD (*n* = 2 data sets; Table 1).

Table 2 shows results from GAA activity assays using nmol/min/g protein units, and glycogen, MuαG, or maltose/ MuαG as a substrate. Three studies reported data from patients with IOPD; values were 0.3–130 nmol/min/g protein (MuαG substrate; *n* = 2 data sets) and 12.5 ± 11.0 nmol/min/g protein (maltose/MuαG substrate; *n* = 1 data set). Three studies reported data from patients with LOPD; values were 8.45 ± 3.22 nmol/min/g protein (glycogen substrate; *n* = 1 data set) and 0.9–8.42 nmol/min/g protein (maltose/MuαG substrate; *n* = 3 data sets; Table 2).

Table 3, Table 4 report data using μM of glucose/min/g protein and mol/g wet-weight muscle units, respectively. Results showed GAA activity in patients with IOPD to be 0.44–2.89 μM of glucose/min/g protein (maltose substrate, *n* = 3 data sets; Table 3), and for patients with LOPD, 0.56–1.03 μM of glucose/min/g protein (maltose substrate, *n* = 3 data sets; Table 3), 0.09 μM of glucose/min/g protein (glycogen substrate, *n* = 1 data set; Table 3), and 0.33 mol/g wet-weight muscle (maltose/MuαG substrate, *n* = 1 data set; Table 3). Three studies reported GAA activity as a percentage of control; assays using glycogen as the substrate reported a mean 0% of control for IOPD and < 0.5% of control for LOPD, while assays using maltose/MuαG reported a median of 16% (15–17%) of control for LOPD (Table 4). There was no clear relationship between GAA activity and muscle biopsied.

**Table 4:** GAA activity and glycogen content (GAA activity unit: of % of control).

Author, Year	Patient number/ biopsy number	Muscle	pH	Assay	GAA activity	
					% of control	
					Median (range)	Test number
Slonim, 2000 [53]	8 (I)/8	Unknown	NA	Glycogen	0	8
	11 (L)/11	Unknown	NA		<0.5	11
Angelini, 2004^†^ [47,48]	2 (L)/2	Q	NA	Maltose/MUαG	16 (15–17)	2

GAA: acid α-glucosidase; I: infantile-onset Pompe disease; L: late-onset Pompe disease; MUαG: 4-methylumbelliferyl α-glucopyranoside; NA: not available; Q: quadriceps.

As with healthy individuals, glycogen content in patients with either IOPD or LOPD was evaluated using only two methods. As expected, healthy individuals had lower glycogen levels in their muscle biopsies compared with patients with Pompe disease. In addition, glycogen content was higher in patients with IOPD compared with LOPD. In patients with IOPD, glycogen content ranged from 0.12% to 369.0% (*n* = 10 data sets; Table 1, Table 2) and 1480 μg/mg protein (*n* = 2 data sets; Table 3). Glycogen content in patients with LOPD was 0.8–5.2% (*n* = 4 data sets; Table 1, Table 2) and 86 μg/mg protein (*n* = 4 data sets; Table 3).

## 4 Discussion

Pompe disease is a rare genetic disorder characterized by a deficiency of GAA, resulting in glycogen accumulation in various tissues, particularly skeletal muscles. Current guidelines state that to make a diagnosis of Pompe disease, GAA enzyme activity should be assessed, a deficiency noted, and the presence of two disease-associated GAA variants should be confirmed using genetic testing [54]. Leukocytes, dried blood spots (DBS), and fibroblasts obtained from skin biopsies are often used for enzymatic diagnostic assays; however, it is recognized that analysis of muscle biopsies is more accurate as this is the tissue at risk [55]. In addition, there is a moderate risk of false results when using leukocyte-based or DBS assays, often necessitating a second assay to confirm a diagnosis [54]. Fibroblast assays require a long culturing time to derive fibroblasts from skin biopsies, making the approach unsuitable for timely diagnosis [54]. Although there are drawbacks to using muscle biopsies (mainly, the invasive nature of obtaining samples), this approach remains efficient in diagnosing Pompe disease. However, the lack of agreed approaches in terms of methodologies, substrates for analysis, and biopsy sites limit the use and comparability of the results. The current review provides a unique historical record and analysis of studies that have quantified GAA activity and glycogen content in muscle biopsies from patients with Pompe disease. It should, however, be noted that due to the low number of data points available, we chose to include data from all studies, regardless of pH.

The findings reported here show that, as expected, GAA activity is higher and glycogen content lower in healthy individuals' muscles compared with patients with Pompe disease [13,56]. Data reported here suggest that GAA activity is markedly lower in patients with IOPD than those with LOPD, as evidenced by increased glycogen – a result observed across all analysis methods. Taken together with other studies examining the severity of both forms of Pompe disease, this suggests that more severe disease (i.e., IOPD) is associated with lower levels of GAA activity. These findings are supported by previous publications that reported an observational trend between disease severity and GAA activity [55,57].

As expected, glycogen content in muscle biopsy specimens was also lower in healthy individuals compared with Pompe disease patients. Furthermore, patients with IOPD had notably higher glycogen content levels in skeletal muscles than those with LOPD. The more pronounced accumulation of glycogen along with lower GAA activity in patients with IOPD (compared with LOPD) is associated with the more severe symptoms in these patients.

Most of the studies in this analysis used biopsies from the quadriceps femoris muscle or unspecified mixed muscle groups, including quadriceps femoris, to quantify GAA activity and glycogen content. However, drawing any conclusions about GAA activity or glycogen content in a particular muscle was not possible, mainly due to the small sample size, variation in data reported, and methodological approaches. Indeed, the major limitation of this work is the wide variation in methodologies, making it difficult to draw meaningful comparisons across all data. As the included publications span over 50 years, “accepted” methods to quantify GAA activity and glycogen content have changed over time. To overcome or limit the impact of such changes, we focused on most commonly used enzymatic methods, in which maltose, glycogen, and MuαG were used as substrates. Despite this, variations still exist in the methods reported. Enzymatic methods used to determine GAA activity were conducted under acidic (pH 4.0–4.5) or neutral (pH 6.5–7.0) conditions in tissue homogenates or protein samples, and glucose concentration was then determined using spectrophotometry or fluorometry. However, there are variations in the specific substrates, their concentrations, and the pH conditions under which the reactions were carried out, even when the same substrate and measurement method were used. The implications of these methodological differences on study outcomes are unknown. Each laboratory has its own assay reference data which is used to confirm or disprove enzyme deficiency in every patient examined. A comprehensive, single-laboratory study of all diagnostic blood-based enzymatic assays conducted over 28-years comparing the MuαG and glycogen substrates suggested that MuαG with fibroblast samples is a more reliable substrate for discerning healthy from diseased individuals, and the only assay that could distinguish between classic IOPD versus childhood or adult onset [58]. However, a similar comparison has not been conducted with muscle biopsies.

The methods used to determine glycogen content levels included amyloglucosidase hydrolysis and the anthrone approaches; both utilize different reagents to quantify glucose released during glycogen hydrolysis. However, the lack of comprehensive method descriptions in some publications limited objective determination and comparison of study results. Another limitation is that the analysis only covers articles written in English. We note that all the limitations underscore the need for a consistent approach and standardization of quantifying GAA enzyme activity and glycogen content in muscle biopsy specimens from healthy individuals and patients living with Pompe disease.

There is a clear unmet need for establishing reference values for muscle GAA activity and glycogen levels to improve comparability between clinical studies and case reviews in this evolving rare disease. To establish a reliable threshold for the percentage of GAA activity that is considered as a “non-disease condition”, a reference range of normal GAA activity measured in limb muscle (e.g., vastus lateralis or biceps brachii) would first need to be defined using a standardized assay. The lack of this information in the literature only serves to strengthen the recommendations provided here.

Ideally, our findings would provide confirmation of the best methods to be used for enzyme assays or glycogen measurement. However, at present, we can only provide a summary of the state-of-art methodology rather than definitive guidance on optimal methodology. Further studies are needed to investigate best methods, such as a large study that uses a single standardized assay to assess GAA activity in numerous muscle biopsies taken from both controls and individuals with Pompe disease.

## 5 Conclusion

As expected, this analysis provides evidence of the relationship between Pompe disease severity and the levels of GAA enzyme activity and glycogen content in muscle. The substantial methodological variations noted here demonstrate the need for globally standardized methods for measuring muscle GAA activity and glycogen content, to allow more accurate assessment of the disease severity and better define the efficacy parameters of a therapeutic intervention.

## Funding

This publication was funded by Astellas Gene Therapies.

## Author contributions

All authors provided a substantial contribution to the conception, design, and interpretation of the work, provided critical review and approval of the final draft. All authors agree to be accountable for the work.

## Author statement

As this was a systemic literature review, approval by an institutional review board and obtaining of informed consent was not required. Generative artificial intelligence (AI) and AI-assisted technologies were not used in the writing process of this manuscript.

## CRediT authorship contribution statement

**Benedikt Schoser:** Writing – review & editing, Writing – original draft, Visualization, Validation, Supervision, Resources, Project administration, Methodology, Investigation, Funding acquisition, Formal analysis, Data curation, Conceptualization. **Nina Raben:** Writing – original draft, Validation, Supervision, Methodology, Formal analysis, Data curation, Conceptualization. **Fatbardha Varfaj:** Writing – original draft, Visualization, Validation, Formal analysis, Data curation. **Mark Walzer:** Writing – review & editing, Validation, Formal analysis. **Antonio Toscano:** Writing – original draft, Validation, Supervision, Methodology, Investigation, Funding acquisition, Formal analysis, Data curation, Conceptualization.

## Declaration of competing interest

B. Schoser reports: advisory board: Amicus, Astellas, Bayer, Maze, Sanofi, Taysha. Unrestricted Contracted Research; Amicus, Astellas. Honoraria; Kedrion, Alexion, Argenx, Spark. Travel Expenses; Astellas, Amicus, Sanofi. N. Raben is an employee of M6P Therapeutics. F. Varfaj reports no competing interests. M. Walzer is an employee of Astellas Gene Therapies. A. Toscano reports no competing interests.

## Data Availability

Data will be made available on request.

## Appendix A Supplementary data

Appendix A Image 1

Appendix B Image 2
