# Supplementary material for: Acid α-glucosidase (GAA) activity and glycogen content in muscle biopsy specimens of patients with Pompe disease: A systematic review
Source: Mol Genet Metab Rep. 2024 Apr 25;39:101085. doi: 10.1016/j.ymgmr.2024.101085 (PMC11064613; doi:10.1016/j.ymgmr.2024.101085)
Supplement: Supplementary file 1 — Appendix A [file mmc1.docx]

## **Appendix A – Supplementary methods**

**Selection process**

The searches and subsequent screening of identified titles were carried out by two reviewers. If disagreements arose, then a third party would reconcile the difference. No attempt was made to contact the corresponding authors of any article that did not contain all the outcome data listed to request information.

The selection process used in this analysis was based on two steps, developed around the eligibility criteria:

- Step 1:
  - The title/abstract review was conducted by two reviewers who worked independently to scan through the search results and select all articles that contained at least one key term in each of the three “buckets”: 1) acid alpha-glucosidase, biopsy, GAA activity, glycogen storage; 2) skeletal muscle, myotubules, vacuoles, biopsy; and 3) acid maltase deficiency, glycogenosis type II, glycogen storage disease type II, Pompe.
  - One reviewer searched all of the original PubMed results, while the second reviewer reviewed half (with the half being randomly selected throughout the search results so there was no bias introduced).
  - Inter-rater reliability was calculated as a percentage: (# of papers identified by Reviewer 2 for inclusion / # of papers identified by Reviewer 1) X 100.
  - The full text articles identified from the initial abstract review were subsequently retrieved and evaluated by two reviewers that worked together to identify articles that related directly to the quantification of glycogen content or GAA enzyme activity in skeletal muscle tissue/cells.
  - For inclusion, studies needed to present data on either GAA enzyme activity or glycogen content in the muscle tissue within the main manuscript or supplemental material. These results did not need to be individually presented, they could be presented as means; however, studies were excluded when authors stated that the analysis had been performed without presenting results.
  - No automation tools were used in the selection process outside of the PubMed search engine, all articles were excluded manually.
  - Case reports, series, or studies that focused on the detailed description of the histopathology of muscle biopsies were included, providing data pertaining to the above criteria were included. Case study data were summarized and categorized in a semi-quantified manner in which the presence or lack of distinct features were utilized for comparative analysis throughout these studies.
  - Studies that described histology solely as a means of diagnosis were eliminated. Publications that presented only histology results that described the four distinct features (1. vacuolated fibers; 2. acid phosphatase; 3. lysosomal glycogen; and 4. extra-lysosomal glycogen) with a substantial portion of explanation (>200 words) were included.
  - Based on the retrieval of >20 articles with GAA enzyme activity/glycogen content measured in patients with Pompe disease, articles that used animal models or in vitro methods in their quantification were eliminated from human muscle tissue eligibility criteria step.
  - At this point, an extensive search of the bibliography of all manuscripts that met the human muscle tissue eligibility criteria was conducted. All references were first screened by title and abstract, then full text if appropriate, and ultimately were included if they met the human muscle tissue eligibility requirements. All articles that were identified and included through the reference search would then undergo a bibliographic search until the reference search yielded 0 results.
  - With >60 articles that met human muscle tissue criteria after the bibliographical search, semi-quantitative histology articles were excluded.
  - Some gray literature, publications that are outside of the traditional peer review process, were included through bibliographical search only. Google search was utilized to find any such reports.
  - The reviewers collaborated on the data retrieval and inclusion criteria for measurements to be assessed as present or not. Initial inter-rater reliability was >97%, with all discrepancies easily resolved. Both reviewers confirmed the data retrieval from each article as correct.
- Step 2:
  - The selected studies from step 1 were screened by one reviewer based on the step 2 selection criteria, including patient numbers, muscle groups biopsied, GAA activity assay, and glycogen content assay.

Eligibility criteria for step 1, included:

- Quantification of GAA enzyme activity or glycogen content in human skeletal muscle
- Publication types: Case reports, case series, clinical trials, observational, repeated measures, diagnostic, and screening studies
- Published in English
- Publications that included the following were excluded:
  - All animal models and in vitro studies in which analysis was performed with cells derived from human muscle tissue
  - Reviews
  - Skeletal muscle biopsies that had histological findings that were performed primarily for diagnostic purposes

Eligibility criteria for step 2 included articles that reported the following data:

- Five or more late-onset Pompe disease patients or studies that reported data from patients with IOPD of any number
- Biopsies from one or more muscle groups from biceps, quadriceps, deltoid, and gastrocnemius
- Baseline (pre-treatment) GAA activity and/or glycogen content values
- GAA activity measured with enzymatic assays using maltose, glycogen, or 4-methylumbelliferyl α-glucopyranoside (MuαG) as substrates
- Glycogen content measured with enzymatic assays, including wet weight and glucose release.
