# Supplementary material for: Acid α-glucosidase (GAA) activity and glycogen content in muscle biopsy specimens of patients with Pompe disease: A systematic review
Source: Mol Genet Metab Rep. 2024 Apr 25;39:101085. doi: 10.1016/j.ymgmr.2024.101085 (PMC11064613; doi:10.1016/j.ymgmr.2024.101085)
Supplement: Supplementary file 2 — Appendix B [file mmc2.docx]

**Appendix B – Supplementary table**

**Table B.1**Summary of all articles included in the analysis.

| **Author, year** | **# of pts** | | **(Total # of biopsy samples included in the study of PD pts): Brief description of study** |
| --- | --- | --- | --- |
|  | **IOPD** | **LOPD** |  |
| Amalfitano, 2001[1] | 3 |  | **(Total of 6 muscle biopsies):** A phase I/II, open-label, single-dose study of rhGAA ERT in 3 IOPD patients. Improvements of skeletal muscle functions and cardiac functions were noted. Muscle biopsies confirmed reductions in glycogen accumulation. (Q) – **USA** |
| Angelini, 2003[2] |  | 11 | **(Total of 11 muscle biopsies):** Clinical and morphological features in 11 LOPD patients with splicing gene mutation. Suggested a predominant role of Golgi in vesicle proliferation and extensive intra-fibral membrane remodeling. (B, D, Ips, Q) –**Italy** |
| Angelini, 2004[3] |  | 11 | **(Total of 11 muscle biopsies):** Natural history and morphological features in 11 LOPD patients. An open prospective clinical trial with a b2 agonist (albuterol) and pulsed branched-chain amino acids in 5 patients for 6 months + 3 years. (B, D, Ips, Q) – **Italy** |
| Busch, 1979[4] | 1 | 1 | **(Total of 2 muscle biopsies):** Case report. The concurrence of IOPD and LOPD in the same family. Case 1: IOPD patient; autopsy performed; Case 2: LOPD – paternal grandfather of case 1. (Q, NS) – **The Netherlands** |
| Engel, 1970[5] | 2 | 4 | **(Total of 9 muscle biopsies):** 4 LOPD cases (and 2 IOPD cases as positive control). The morphological and biochemical findings (glycogen, GAA) showed an excess of muscle glycogen. (A, G, Q, T, Th) – **USA** |
| Kishnani, 2006[6] | 8 |  | **(A minimum of 12 muscle biopsies):** An open-label study examining the safety and efficacy of ERT in treatment of IOPD. 8 IOPD patients were enrolled in the 52-week initial phase + extension phase (~153 weeks) of ERT. GAA activity and glycogen content (histomorphometric) were measured before and after ERT started. (Q) – **USA and Europe** |
| Kishnani, 2007[7] | 18 |  | **(Total of 54 muscle biopsies):** A multinational, multicenter, open-label, dose-ranging study examining the safety and efficacy of ERT in a large cohort of severe IOPD patients who began treatment prior to 6 months of age. Muscle biopsies performed at baseline, week 12, and 52. 54 specimens collected, 36 were reported. (Q) – **USA, Europe, Taiwan, Israel** |
| Klinge, 2005[8] | 2 |  | **(Total of 4 muscle biopsies):** A 10-month follow-up study in 2 IOPD patients that previously completed a 48-week course of ERT (the phase II trial in Klinge, 2005 b). (Q) – **Germany**  *Same cases and data set as Klinge, 2005b* |
| Klinge, 2005[9] | 2 |  | **(Total of 10 muscle biopsies):** A phase II, open-label, single-center, single-dose clinical trial including 2 IOPD patients, receiving ERT over a period of 48 weeks by weekly infusions of rhGAA 40 mg/kg. Open muscle biopsies were taken from quadriceps muscle at week 0, 12, 24, 36, and 48. An overall improvement was observed. (Q) – **Germany** |
| Koeberl, 2018[10] |  | 11 | **(Total of 22 muscle biopsies):** A phase I/II double-blind, randomized, placebo-controlled clinical trial of clenbuterol in LOPD. 11 of 13 patients completed the study. (Q) – **USA** |
| Koster, 1978[11] | 1 | 1 | **(Total of 3 muscle biopsies):** Case series of 1 IOPD patient (autopsy) and 1 LOPD patient (the paternal grandfather of case 1, 2 biopsy specimens) concurrent in 1 family. (D, Q, NS) – **The Netherlands** |
| Loonen, 1981[12] | 1 | 1 | **(Total of 2 muscle biopsies):** Case series of a family with different clinical forms of PD (1 IOPD patient - 3rd generation, and 1 LOPD patient - 1st generation). Describes the clinical, enzymatic, and genetic studies in the 2 patients and their relatives. (Q, To, NS) – **The Netherlands**  *Also reported in Koster 1978, Busch 1979, and Hoefsloot 1990* |
| Mekanik, 1966[13] | 1 |  | **(Total of 3+ muscle biopsies):** Case study: 1 IOPD patient. Glycogen accumulation and GAA deficiency were noticed in biopsy and autopsy specimens including skeletal muscles. (G) – **USA** |
| Nicolino, 2009[14] | 19 |  | **(Total of 50 muscle biopsies):** An open-label, multicenter study of ERT in 21 infant (3-43 m) PD patients. Patients received IV alglucosidase alfa every 2 weeks up to 168 weeks. Baseline (19 pts) and week 52 (18 pts) biopsies were obtained. Glycogen content test was done at baseline and 52 weeks of treatment (13 pts had repeat biopsies). (Q) – **USA, Europe, Israel** |
| Pellegrini, 1978[15] | 1 |  | **(Total of 3 muscle biopsies):** Histological, histochemical, and ultrastructural examination on muscle tissue in the parents of a PD patient. The muscle biopsy from the patient showed increased glycogen content and absent acid maltase. (B) – **Italy** |
| Pongratz, 1976[16] | 1 |  | **(Total of 2 muscle biopsies):** Case report. 1 IOPD patient with mild myopathy. 2 muscle biopsies were obtained at 10 and 11 months of age. (G) – **Germany** |
| Read, 2001[17] |  | 5 | **(Total of 9 muscle biopsies):** Case series. 5 LOPD patients. Muscle biopsies were obtained from 4 patients. (D, I, Q, Ti, Tr) – **New Zealand** |
| Ripolone, 2018[18] |  | 18 | **(Total of 36 muscle biopsies):** 18 LOPD patients, pre- and post- ERT to survey how ERT can influence skeletal muscle pathobiology and correlation with clinical outcomes (B, D, Q) – **USA** |
| Slonim, 2000[19] | 8 | 11 | **(Total of 19 muscle biopsies):** 2 subtypes of IOPD patients -11/12 non typical, 8/10 typical underwent muscle biopsy. Findings on LM, histochemistry, and EM of muscle were identical across the groups. GAA enzyme activity was undetectable in typical infants, whereas traces of activity were detected in nontypical infants. (Q) – **USA** |
| Slonim, 2006[20] | 2 |  | **(Total of 3 muscle biopsies):** Case report. 2 siblings with IOPD that underwent nutritional and exercise therapy No change of activity between pre- and post- NET. (Q) – **USA** |
| van den Hout, 2001[21] | 4 |  | **(Total of 12 muscle biopsies):** The results of the first 36 weeks of a single-center, open-label pilot study on ERT in 4 IOPD patients with recombinant human a-glucosidase produced in the milk of transgenic rabbits. (Q) – **The Netherlands**  *Same cases were reported in van den Hout 2000 (same biopsies) and van den Hout 2004.* |
| van den Hout, 2004[22] | 4 |  | **(Total of 16 muscle biopsies):** A follow-up study to <3 years of a single-center, open-label pilot study on ERT treatment in 4 IOPD patients. (Q) – **The Netherlands**  *Same cases were reported in van den Hout 2000 and van den Hout 2001.* |
| Verity, 1991[23] | 1 |  | **(Total of 1 muscle biopsy):** Case report of 1 patient with typical IOPD and lipid storage myopathy. (Q) – **USA** |
| Zellweger, 1965[24] | 1 | 1 | **(Total of 4 muscle biopsies):** Case series of a mild form of AMD in 2 brothers with clinical manifestation limited to the skeletal muscles. Case 1: IOPD with mild symptoms; Case 2: LOPD. (G, Pn, Ti) – **USA** |

#: number; EM: electron microscopy;AMD: acid maltase deficiency; ERT: enzyme replacement therapy; GAA: acid α-glucosidase; IOPD: infantile-onset Pompe disease; LM: light microscopy; LOPD: late-onset Pompe disease; NS: not specified; PD: Pompe disease; pts.: patients; ref: reference; rhGAA: recombinant human GAA.
Muscle groups: Ab: abdominus, B: bicep, D: deltoid, Di: diaphragm, G: gastrocnemius, I: intercostal, Ips: iliopsoas, Is: infraspinatus, L: larynx, P: pectoralis, Pn: pennate, Ps: psoas, Q: quadricep, T: triceps, Te: temporalis, Th: thigh, Ti: tibialis, To: tongue, Tr: trapezius, Sp: spinae.

When 2 or more articles from the same first author, published in the same year, were listed in the summary tables, letters in alphabetical order were added after the year of publication to distinguish the different articles.

**References**

[1] A. Amalfitano, A.R. Bengur, R.P. Morse, J.M. Majure, L.E. Case, D.L. Veerling, J. Mackey, P. Kishnani, W. Smith, A. McVie-Wylie, J.A. Sullivan, G.E. Hoganson, J.A. Phillips, 3rd, G.B. Schaefer, J. Charrow, R.E. Ware, E.H. Bossen, Y.T. Chen, Recombinant human acid alpha-glucosidase enzyme therapy for infantile glycogen storage disease type II: results of a phase I/II clinical trial, Genet Med. 3 (2001) 132-138. <https://www.ncbi.nlm.nih.gov/pubmed/11286229>.

[2] C. Angelini, G. Cenacchi, A.C. Nascimbeni, L. Fulizio, Morphological changes in late onset acid Maltase deficient patients with splicing gene mutation, Acta Myol. 22 (2003) 90-96. <https://www.ncbi.nlm.nih.gov/pubmed/15088498>.

[3] C. Angelini, E. Pegoraro, S. Zambito Marsala, L. Vergani, A.C. Nascimbeni, L. Fulizio, M. Fanin, Adult Acid Maltase Deficiency: an Open Trial with Albuterol and Branched-Chain Amino Acids, Basic Appl Myol. 14 (2004) 71-78.

[4] H.F. Busch, J.F. Koster, T.W. van Weerden, Infantile and adult-onset acid maltase deficiency occurring in the same family, Neurology. 29 (1979) 415-416. <https://www.ncbi.nlm.nih.gov/pubmed/377133>.

[5] A.G. Engel, Acid maltase deficiency in adults: studies in four cases of a syndrome which may mimic muscular dystrophy or other myopathies, Brain. 93 (1970) 599-616. <https://www.ncbi.nlm.nih.gov/pubmed/4918728>.

[6] P.S. Kishnani, M. Nicolino, T. Voit, R.C. Rogers, A.C. Tsai, J. Waterson, G.E. Herman, A. Amalfitano, B.L. Thurberg, S. Richards, M. Davison, D. Corzo, Y.T. Chen, Chinese hamster ovary cell-derived recombinant human acid alpha-glucosidase in infantile-onset Pompe disease, J Pediatr. 149 (2006) 89-97. <https://www.ncbi.nlm.nih.gov/pubmed/16860134>.

[7] P.S. Kishnani, D. Corzo, M. Nicolino, B. Byrne, H. Mandel, W.L. Hwu, N. Leslie, J. Levine, C. Spencer, M. McDonald, J. Li, J. Dumontier, M. Halberthal, Y.H. Chien, R. Hopkin, S. Vijayaraghavan, D. Gruskin, D. Bartholomew, A. van der Ploeg, J.P. Clancy, R. Parini, G. Morin, M. Beck, G.S. De la Gastine, M. Jokic, B. Thurberg, S. Richards, D. Bali, M. Davison, M.A. Worden, Y.T. Chen, J.E. Wraith, Recombinant human acid [alpha]-glucosidase: major clinical benefits in infantile-onset Pompe disease, Neurology. 68 (2007) 99-109. <https://www.ncbi.nlm.nih.gov/pubmed/17151339>.

[8] L. Klinge, V. Straub, U. Neudorf, T. Voit, Enzyme replacement therapy in classical infantile pompe disease: results of a ten-month follow-up study, Neuropediatrics. 36 (2005) 6-11. <https://www.ncbi.nlm.nih.gov/pubmed/15776317>.

[9] L. Klinge, V. Straub, U. Neudorf, J. Schaper, T. Bosbach, K. Gorlinger, M. Wallot, S. Richards, T. Voit, Safety and efficacy of recombinant acid alpha-glucosidase (rhGAA) in patients with classical infantile Pompe disease: results of a phase II clinical trial, Neuromuscul Disord. 15 (2005) 24-31. <https://www.ncbi.nlm.nih.gov/pubmed/15639117>.

[10] D.D. Koeberl, L.E. Case, E.C. Smith, C. Walters, S.O. Han, Y. Li, W. Chen, C.P. Hornik, K.M. Huffman, W.E. Kraus, B.L. Thurberg, D.L. Corcoran, D. Bali, N. Bursac, P.S. Kishnani, Correction of Biochemical Abnormalities and Improved Muscle Function in a Phase I/II Clinical Trial of Clenbuterol in Pompe Disease, Mol Ther. 26 (2018) 2304-2314. <https://www.ncbi.nlm.nih.gov/pubmed/30025991>.

[11] J.F. Koster, H.F. Busch, R.G. Slee, T.W. Van Weerden, Glycogenosis type II: the infantile- and late-onset acid maltase deficiency observed in one family, Clin Chim Acta. 87 (1978) 451-453. <https://www.ncbi.nlm.nih.gov/pubmed/28188>.

[12] M.C. Loonen, A.W. Schram, J.F. Koster, M.F. Niermeijer, H.F. Busch, J.J. Martin, B. Brouwer-Kelder, W. Mekes, R.G. Slee, J.M. Tager, Identification of heterozygotes for glycogenosis 2 (acid maltase deficiency), Clin Genet. 19 (1981) 55-63. <https://www.ncbi.nlm.nih.gov/pubmed/7006871>.

[13] G. Mekanik, R.L. Smith, R.M. MacLeod, Enzyme patterns in glycogen storage disease type II (Pompe's disease), Metabolism. 15 (1966) 641-648. <https://www.ncbi.nlm.nih.gov/pubmed/4288232>.

[14] M. Nicolino, B. Byrne, J.E. Wraith, N. Leslie, H. Mandel, D.R. Freyer, G.L. Arnold, E.K. Pivnick, C.J. Ottinger, P.H. Robinson, J.C. Loo, M. Smitka, P. Jardine, L. Tato, B. Chabrol, S. McCandless, S. Kimura, L. Mehta, D. Bali, A. Skrinar, C. Morgan, L. Rangachari, D. Corzo, P.S. Kishnani, Clinical outcomes after long-term treatment with alglucosidase alfa in infants and children with advanced Pompe disease, Genet Med. 11 (2009) 210-219. <https://www.ncbi.nlm.nih.gov/pubmed/19287243>.

[15] G. Pellegrini, G. Mosca, C. Cerri, Pompe's disease: ultrastructural alterations of muscle tissue in parents, Acta Neurol Scand. 57 (1978) 216-222. <https://www.ncbi.nlm.nih.gov/pubmed/276244>.

[16] D. Pongratz, I. Schlossmacher, C. Koppenwallner, G. Hubner, An especially mild myopathic form of glycogenosis type II. Problems of clinical and light microscopic diagnosis, Pathol Eur. 11 (1976) 39-44. <https://www.ncbi.nlm.nih.gov/pubmed/132627>.

[17] K. Read, D. Hutchinson, A. Veale, N. Anderson, G. Hammond-Tooke, A. Macfie, Acid maltase deficiency: clinical and laboratory features of adult-onset cases, N Z Med J. 114 (2001) 406-409. <https://www.ncbi.nlm.nih.gov/pubmed/11665929>.

[18] M. Ripolone, R. Violano, D. Ronchi, S. Mondello, A. Nascimbeni, I. Colombo, G. Fagiolari, A. Bordoni, F. Fortunato, V. Lucchini, S. Saredi, M. Filosto, O. Musumeci, P. Tonin, T. Mongini, S. Previtali, L. Morandi, C. Angelini, M. Mora, M. Sandri, M. Sciacco, A. Toscano, G.P. Comi, M. Moggio, Effects of short-to-long term enzyme replacement therapy (ERT) on skeletal muscle tissue in late onset Pompe disease (LOPD), Neuropathol Appl Neurobiol. 44 (2018) 449-462. <https://www.ncbi.nlm.nih.gov/pubmed/28574618>.

[19] A.E. Slonim, L. Bulone, S. Ritz, T. Goldberg, A. Chen, F. Martiniuk, Identification of two subtypes of infantile acid maltase deficiency, J Pediatr. 137 (2000) 283-285. <https://www.ncbi.nlm.nih.gov/pubmed/10931430>.

[20] A.E. Slonim, L. Bulone, J. Minikes, A.P. Hays, S. Shanske, S. Tsujino, S. DiMauro, Benign course of glycogen storage disease type IIb in two brothers: nature or nurture?, Muscle Nerve. 33 (2006) 571-574. <https://www.ncbi.nlm.nih.gov/pubmed/16320310>.

[21] J.M. Van den Hout, A.J. Reuser, J.B. de Klerk, W.F. Arts, J.A. Smeitink, A.T. Van der Ploeg, Enzyme therapy for pompe disease with recombinant human alpha-glucosidase from rabbit milk, J Inherit Metab Dis. 24 (2001) 266-274. <https://www.ncbi.nlm.nih.gov/pubmed/11405345>.

[22] J.M. Van den Hout, J.H. Kamphoven, L.P. Winkel, W.F. Arts, J.B. De Klerk, M.C. Loonen, A.G. Vulto, A. Cromme-Dijkhuis, N. Weisglas-Kuperus, W. Hop, H. Van Hirtum, O.P. Van Diggelen, M. Boer, M.A. Kroos, P.A. Van Doorn, E. Van der Voort, B. Sibbles, E.J. Van Corven, J.P. Brakenhoff, J. Van Hove, J.A. Smeitink, G. de Jong, A.J. Reuser, A.T. Van der Ploeg, Long-term intravenous treatment of Pompe disease with recombinant human alpha-glucosidase from milk, Pediatrics. 113 (2004) e448-457.

[23] M.A. Verity, Infantile Pompe's disease, lipid storage, and partial carnitine deficiency, Muscle Nerve. 14 (1991) 435-440. <https://www.ncbi.nlm.nih.gov/pubmed/1870635>.

[24] H. Zellweger, B.I. Brown, W.F. McCormick, J.B. Tu, A mild form of muscular glycogenosis in two brothers with alpha-1, 4-glucosidase deficiency, Ann Paediatr. 205 (1965) 413-437. <https://www.ncbi.nlm.nih.gov/pubmed/5217754>.
